# Accuracy of a patient-specific 3D-printed drill guide for placement of bicortical screws in atlantoaxial ventral stabilization in dogs

**DOI:** 10.1371/journal.pone.0272336

**Published:** 2022-08-01

**Authors:** Yong Yu, Jinsu Kang, Namsoo Kim, Suyoung Heo

**Affiliations:** Department of Veterinary Surgery, Jeonbuk National University, Gobong-ro, Iksan, South Korea; Rush University Medical Center, UNITED STATES

## Abstract

Atlantoaxial instability (AAI) in dogs refers to abnormal motion at the C1–C2 articulation due to congenital or developmental anomalies. Surgical treatment options for AAI include dorsal and ventral stabilization techniques. Ventral stabilization techniques commonly utilize transarticular and vertebral body screws or pins. However, accurate screw insertion into the vertebrae of C1 and C2 is difficult because of the narrow safety corridors. This study included 10 mixed dogs, 1 Pomeranian, and 1 Shih-Tzu cadaver. All dogs weighed <10 kg. Each specimen was scanned using computed tomography (CT) from the head to the 7th cervical vertebrae. This study used 12 bone models and 6 patient-specific drill guides. Bone models were made using CT images and drill guides were created through a CAD (computer-aided design) program. A total of six cortical screws were used for each specimen. Two screws were placed at each of the C1, C2 cranial, and C2 caudal positions. Postoperative CT images of the cervical region were obtained. The degree of cortex breaching and angle and bicortical status of each screw was evaluated. The number of screws that did not penetrate the vertebral canal was higher in the guided group (33/36, 92%) than in the control group (20/36, 56%) (P = 0.003). The screw angles were more similar to the reference angle compared to the control group. The number of bicortically applied screws in the control group was 28/36 (78%) compared to 34/36 (94%) in the guided group. Differences between the preoperative plan and the length of the applied screw at the C1 and C2 caudal positions were determined by comparing the screw lengths in the guide group. The study results demonstrated that the use of a patient-specific 3D-printed drill guide for AAI ventral stabilization can improve the accuracy of the surgery. The use of rehearsal using bone models and a drilling guide may improve screw insertion accuracy.

## Introduction

Atlantoaxial instability (AAI) in dogs refers to the abnormal motion at the C1–C2 articulation due to congenital or developmental anomalies of the dens and supporting ligaments that are typically exacerbated by trauma. The resulting atlantoaxial subluxation (AAS) can lead to spinal cord compression [1,2]. AAI occurs most commonly in small breed dogs although traumatic AAS due to dens fracture or ligament rupture can occur in dogs of any breed.

AAS can be diagnosed using plain cervical vertebral column radiographs. The space between the dorsal laminae of the atlas and the spinous process of the axis is visualized with head and neck flexion; however, this maneuver can be hazardous. AAS can also be more safely diagnosed via the overlap of the atlas and axis, dens/C2 ratio, and C1–C2 angle [3]. Diagnosis via computed tomography (CT) and magnetic resonance imaging (MRI) is preferable to a stressed radiographic view, and is safer. CT can assist in deciding the appropriate size of implants and surgical implant placement pathway.

AAI can be conservatively or surgically managed for stabilization. A previous study reported a 62.5% success rate of conservative therapy, but with the risk of clinical sign recurrence and worsening [4]. Surgical treatment of AAI includes ventral and dorsal stabilization techniques but with risks for iatrogenic injury. Ventral stabilization techniques are commonly preferred because they avoid the risk of dorsal wiring and allow atlantoaxial junction visualization and arthrodesis techniques including cancellous bone graft placement [5]. The reported implants for ventral stabilization include screws, threaded pins, or Kirschner wires (K-wires), supported by polymethylmethacrylate (PMMA) or plates [5,6].

Accurate screw insertion into the C1 and C2 vertebrae are difficult because of the narrow safety corridor, and the risk of bone fractures and iatrogenic damage to the spinal cord, blood vessels, and nerves. The reported success rate of ventral stabilization varies from 47% to 92%, and the perioperative mortality rate has been between 4% and 30% in dogs [6–11]. To reduce the risks, studies of the safety corridor have been conducted, and the use of patient-specific three-dimensional (3D) printed drill guides has been reported [12–15]. Custom-made implants using 3D printers have a positive effect on surgical time, patient recovery time, and surgical success [16]. Two recent case series of AAI surgery that uses patient-specific guides for ventral stabilization have shown high success rates (83.3% and 93%) in a total of 30 canine patients [14,15].

Toni et al. (2020) evaluated the accuracy of screw placement using postoperative CT and reported high accuracy. Of 61 bicortical screws placed, 57 (93%) were fully contained within the pedicle and vertebral body and four (7%) partially breached the medial pedicle wall. However, to our understanding, no studies have evaluated the accuracy of drill guides compared to placement by eye using only anatomical landmarks at C1/2 in dogs. Accuracy comparisons of customized drill guides in other vertebral locations indicated a high accuracy in the guide groups [17–19].

This study aimed to compare the accuracy of screw placement in C1 and C2 using a patient-specific drill guide versus freehand drilling for ventral stabilization of AAI and evaluate the significance of using 3D-printed models in preoperative planning. The authors hypothesized that (1) the use of a guide in AAI ventral stabilization surgery would provide higher accuracy, and (2) surgical accuracy would be improved if the simulation was performed using a bone model during preoperative planning.

## Materials and methods

### Ethics statement

All dogs were euthanized for medical reasons unrelated to this study and were donated for research purposes by their owners. This study was performed under the approval of the Institutional Animal Care and Use Committee of Jeonbuk National University(Approval number: JBNU 2021–0106). All procedures were performed in accordance with the guidelines regulating animal use and ethics at Jeonbuk National University.

### Cadaveric specimens

This study included 10 mixed dogs, 1 Pomeranian, and 1 Shih-Tzu cadaver (Table 1). All dogs weighed < 10 kg (median, 6.45 kg; range, 2.25–9 kg). Cadavers were stored at −20°C and thawed at room temperature for 24 h before CT scanning and surgery. Cadavers were randomly categorized into two groups as follows: (1) freehand screw insertion group without guides (control group) and (2) screw insertion group with patient-specific 3D-printed drill guides (guide group).

**Table 1:** Signalments of the cadavers and screw sizes.

Cadaver	Breeds	Sex	Weight (kg)	Screw sizes (mm)	Groups
**1**	Mixed	Female	8.7	C1: 2.4	Guide group
				C2 cranial: 2.4	
				C2 caudal: 2.0	
**2**	Pomeranian	Male	2.25	C1: 1.2	Control group
				C2 cranial: 1.2	
				C2 caudal: 1.2	
**3**	Mixed	Female	4.85	C1: 1.5	Control group
				C2 cranial: 1.5	
				C2 caudal: 1.2	
**4**	Mixed	Female	4.6	C1: 2	Guide group
				C2 cranial: 2	
				C2 caudal: 2	
**5**	Mixed	Female	6.65	C1: 2	Control group
				C2 cranial: 2	
				C2 caudal: 1.5	
**6**	Mixed	Male	5.8	C1: 1.5	Guide group
				C2 cranial: 1.5	
				C2 caudal: 1.5	
**7**	Mixed	Male	7	C1: 2	Guide group
				C2 cranial: 2	
				C2 caudal: 2	
**8**	Mixed	Female	7.9	C1: 2	Control group
				C2 cranial: 2	
				C2 caudal: 1.5	
**9**	Mixed	Female	6.6	C1: 2	Control group
				C2 cranial: 2	
				C2 caudal: 1.5	
**10**	Mixed	Male	7.8	C1: 2.4	Guide group
				C2 cranial: 2.4	
				C2 caudal: 2	
**11**	Shih-tzu	Female	6.2	C1: 1.5	Guide group
				C2 cranial: 1.5	
				C2 caudal: 1.5	
**12**	Mixed	Male	9	C1: 2.4	Control group
				C2 cranial: 2.4	
				C2 caudal: 2	

### Computed tomographic (CT) imaging and bone model reconstruction

Each specimen was subjected to CT scans from the head to the 7th cervical vertebrae. CT images were obtained using a 16-slice multi-detector CT scanner (Alexion, TSX-034A, Toshiba Medical System, Tochigi, Japan) with the following parameters: 120 kVp, 150 mAs, 0.688 pitch, 0.75 rotation time, and 1 mm slice thickness using a bone algorithm. Images were stored in DICOM (Digital Imaging and Communications in Medicine) format and imported into 3D slicer software (3D slicer, National Alliance for Medical Image Computing, Boston, MA) for C1-C2 segment bone model making. The bone model was stored in Stereolithography file format (STL). Twelve bone models were printed using a fused deposition modeling (FDM) 3D printer (Replicator +, MakerBot Industries, Brooklyn, USA). The materials used in the bone model were polylactic acid (PLA) (MakerBot Industries) with 0.4 mm nozzle size, 0.1 mm layer thickness, 20% infill density, and 95 mm/s print speed.

### Surgical planning and patient-specific 3D-printed drill guide construction

**Fig 1:**
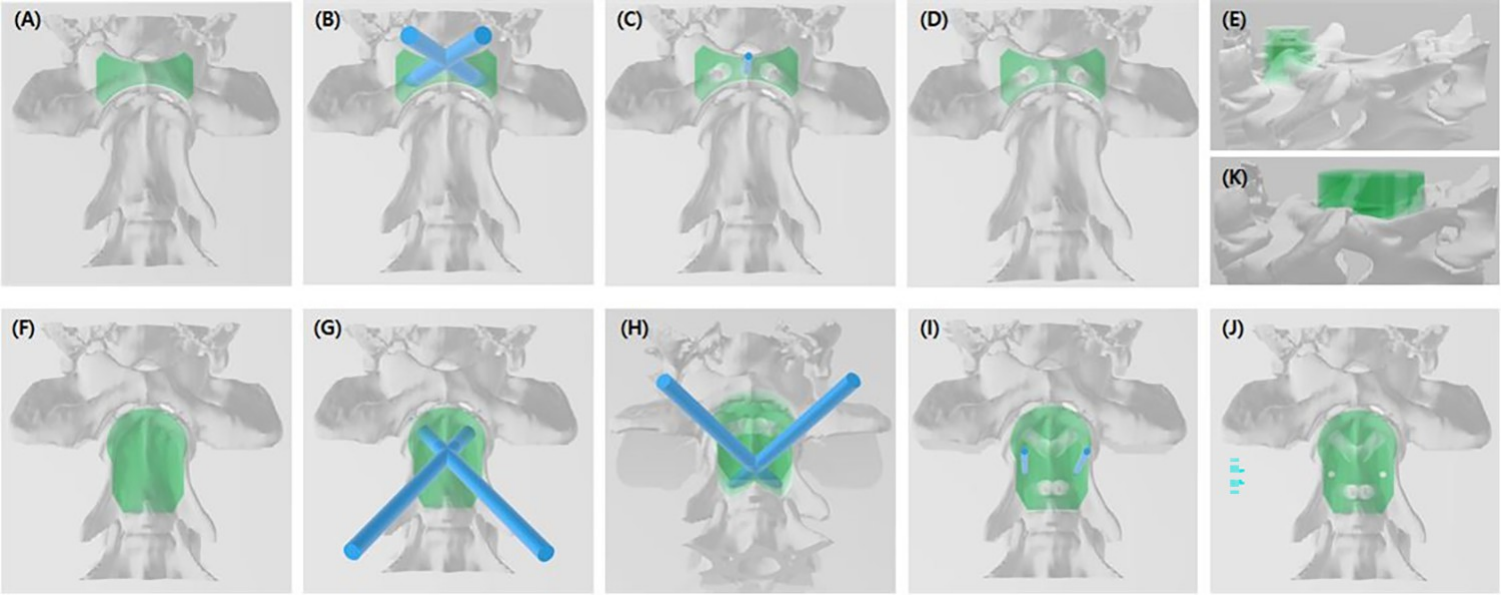
The manufacturing process of the patient-specific guide based on the bone model. Drill guide template for C1 (A) and C2 (F) before making holes. Each hole with a diameter similar to the drill sleeve (B, G and H). A temporary pinhole in the center of the C1 guide, and (C) two temporary pin holes on both sides of the C2 body (I). Completed design of the C1 (D and E) and C2 (J and K) patient-specific drill guide.

**Fig 2:**
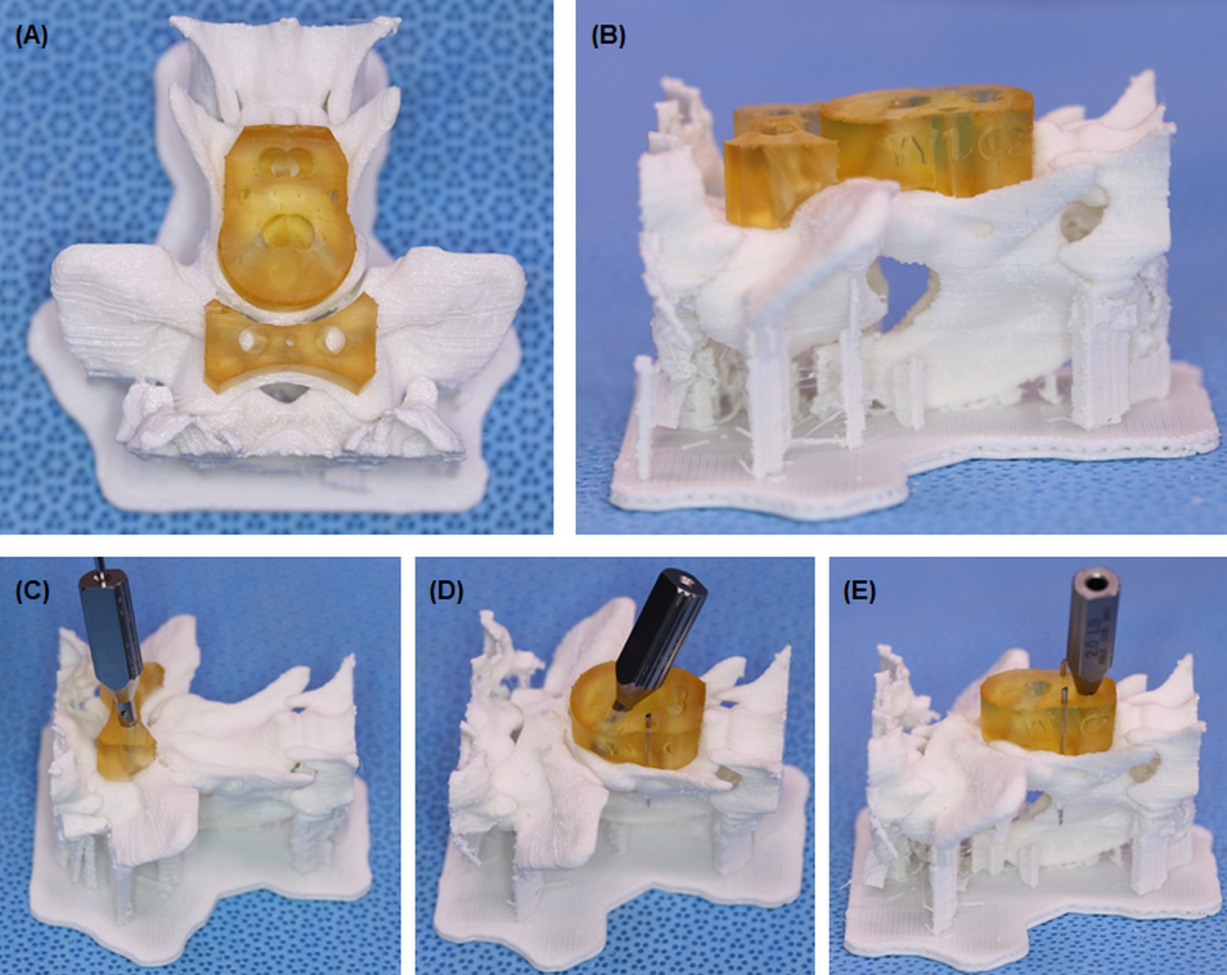
The C1 and C2 guides on the 3D-printed bone model and the drill sleeves. Dorsal view (A) and lateral view (B) of the patient-specific guides on the bone model. The drill sleeve at the C1 guide hole (C). (D) The drill sleeve at the cranial hole of the C2 guide. (E) The drill sleeve at the caudal hole of the C2 guide.

A total of six cortical screws were used for each specimen. Two screws were placed at each of the C1, C2 cranial, and C2 caudal positions [6,15] (Figs 1 and 2). The screws (Able, Jeonbuk, Korea) were made of stainless steel, and 1.2, 1.5, 2.0, or 2.4 mm (thread diameter) screws were used depending on the patient’s size. Each screw angle and insertion position was set according to the reported optimal safe implantation corridors [12,13]. The angle of the C1 screw was set to 20° to the lateral and 10° to the caudal, the C2 cranial screw at 45° to the lateral and 35° to the ventral, and the C2 caudal screw at 1° to the cranial and 29° to the lateral. The estimated screw length was measured in the CT transverse plane. C1 length was calculated as the transverse plane measurement length divided by cos10, C2 cranial length was the transverse plane measurement length divided by cos35, and C2 caudal length was the transverse plane measurement length. The screws stuck out 1–2mm from the far cortex for clinical application, and a length of 3–5 mm was added to the measurement length so that PMMA could be applied.

Drill guide templates were created using a computer-aided design (CAD) software program (3D builder, Microsoft Corporation, Redmond, WA). The C1 and C2 guides were individually produced based on the bone model STL files (Fig 1). Each guide was designed to be fixed to the bone using K-wires. The C1 guide used one K-wire and the C2 guide used two K-wires (Fig 1C and 1I). The K-wires ensured a tight fit of the drill guides on the vertebrae before predrilling the screw holes, thus reducing the possibility of slippage between the guide and the vertebral surface during the drilling process. The diameter of each drill hole was determined to fix the drill sleeve corresponding to the drill bit size to be used (Fig 2). After aligning each vertebra with the sagittal plane, C1 and C2 vertebrae ventral processes were used as reference points. The angle of the hole was set to the previously mentioned values.

The C1 guide was made to fit the ventral tubercle and ventral cortex and was narrower than the transverse foramen on both sides. The C1 vertebrae ventral surface was narrow because most patients with AAI were small. Thus, a temporary pinhole was made in the center of the guide for it to be fixed to the ventral tubercle, which is the thickest part of the C1 ventral surface. The C2 guide was made to fit the body of the C2 and the articular surface of the cranial and ventral crest of the caudal. Temporary pinholes were designed to not invade the vertebral canal on either side of the C2 body. The guides were 3D-printed using a resin 3D-printer (Pixel one, Zerone, Gyeonggi, Korea), and dental surgical guide resin (SG-100, Graphy, Seoul, Korea) was used. The layer thickness was set to 50 μm, and the cure time was set to 5.5 s. After printing, the guides were washed, dried for 30 min, and UV-light cured at a wavelength of 405 nm for 60 min (3DP-100S, CUBICON, Gyeonggi, Korea).

The manufacturing time of the patient-specific guide in the CAD program approximately took 2 h and the printing time of the guide using a 3D- printer was 4 h. After printing 12 bone models and 6 guides, simulated surgeries were performed on the bone models, with (6) or without (6) a drill guide. Holes were made and the depth of each hole was measured. The lengths of the used screws in the bone model were also measured.

### Surgical procedure

All dogs were positioned in dorsal recumbence with the neck extended, thoracic limbs extended, and secured caudally. A towel was placed at the bottom of the neck to elevate the joints of C1 and C2. The wing of C1 was palpated, and the ventral tubercle was positioned in the center and secured using a vacuum bag. A ventral midline approach that transects the right sternothyroideus muscle was used. The trachea and larynx were retracted to the left. The longus colli muscles and their insertion on C1 were elevated and retracted. The C1–C2 joint was exposed, and the synovial membrane was incised. The soft tissues present on the ventral side of C1 and C2 were removed as cleanly as possible using a periosteal elevator.

**Fig 3:**
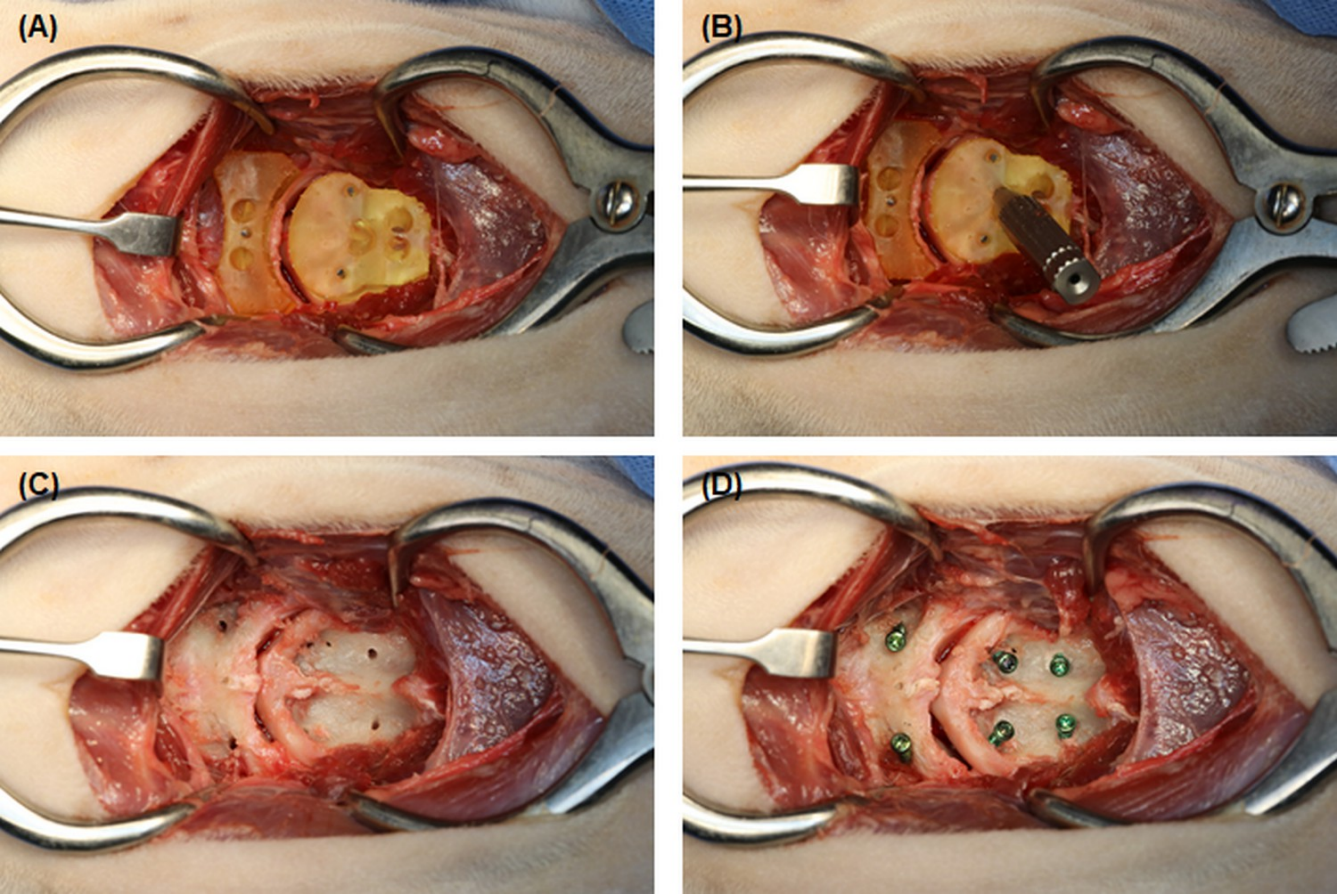
Application of the patient-specific guide to the cadaver. (A) Patient-specific guide of the C1 and C2 and the temporary pins inserted. (B) The drill sleeve on the patient-specific guide and the creation of a drill tract using a drilling. (C) Removal of the guide and temporary pins. (D) The surgical site with the inserted screws 3–5 mm above the bone.

In the control group, holes were made in C1 and C2 using a drill guide (Able) placed by the eye regarding the bone models. After measuring the length using a depth gage, a screw (of pre-planned diameter) was inserted. In the guide group, holes were created using a patient-specific 3D-printed drill guide (Fig 3). After placing a patient-specific guide on the ventral cortex of C1, a temporary pin was inserted. The drill sleeve was placed on the patient-specific guide, and a drilling tract was bicortically created using a drill of appropriate size. The guide and the temporary pin were sequentially removed. After measuring the length using a depth gage, a screw (of pre-planned diameter) was inserted. A patient-specific guide was placed on the body of C2 and fixed to fit the articular surface and ventral crest. Two temporary pins were inserted. Holes were created in the same way as C1 and inserted screws. All screws were left exposed 3–5 mm above the bone to secure the bone cement. Routine closure was performed, including right sternothyroideus muscle repair.

### Post-surgical evaluation

**Fig 4:**
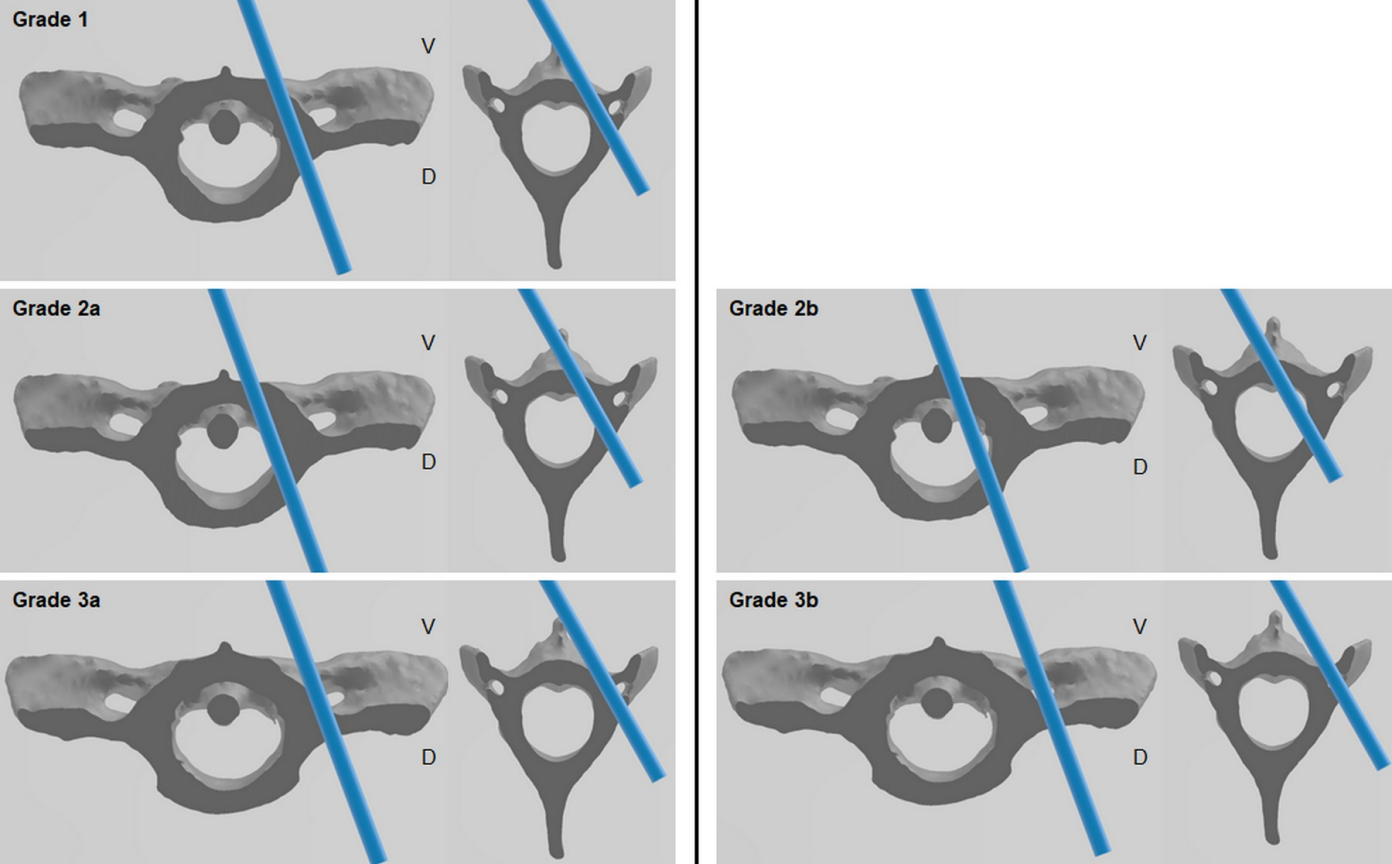
Transverse images of C1 and C2 with pedicle screw trajectories in place. Grading of the screw position by Zdichavsky modified classification. Grade 1: fully contained within the pedicle and vertebral body. Grade 2a: medial pedicle wall penetration. Grade 2b: entirely medial to the pedicle wall. Grade 3a: partial lateral breaching. Grade 3b: full lateral breaching. Lateral breaching was defined as an intrusion into the C1 transverse foramen or C2 transverse foramen and breach of the C2 lateral cortex.

Postoperative CT images of the cervical region were obtained using the previously mentioned protocol. CT images of the transverse, sagittal, and dorsal planes were used to evaluate the degree of screw penetration of the adjacent pedicle cortex. The degree of cortex breaching was subjectively evaluated using the modified Zdichavsky classification as follows (Fig 4) [20]. Grade 1: the screw was fully contained within the pedicle and vertebral body. Grade 2a: the screw penetrated the medial pedicle wall. Grade 2b: the screw was entirely medial to the pedicle wall, so the vertebral canal was penetrated. Grade 3a: the screw was partially breaching the lateral cortex. Grade 3b: the screw was fully breaching the lateral cortex. Additionally, lateral breaching was further defined as an intrusion into the C1 transverse foramen or C2 transverse foramen or a breach of the C2 lateral cortex.

**Fig 5:**
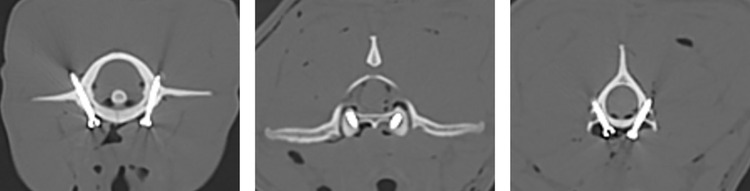
Transverse CT image of C1, C2 cranial, and C2 caudal.

The angle of each screw was measured using the previously published methods [12,13]. The angle of each screw was evaluated using CT images based on the screw insertion location. The C1 angle was measured through the transverse and sagittal planes, the C2 cranial angle through the dorsal and sagittal planes, and the C2 caudal angle through the sagittal and transverse planes. The C1 and C2 ventral processes, the C1 dorsal tubercle, and the C2 spinous process were used as a point of baseline. The bicortical status of each screw (whether two cortices were engaged by the screw) was evaluated using the CT transverse plane, and only those that had penetrated the outer cortex of 1 mm or more were included (Fig 5).

### Statistical methods

The statistical analysis was performed using the Statistical Package for the Social Sciences (version 26.0; IBM, Armonk, New York). The statistical analysis was conducted on four values as follows: (1) grade evaluation, (2) screw insertion angles, (3) bicortical status, and (4) screw length comparison using CT, bone models, and cadavers in the guide group. The Mann–Whitney test was used to compare the grade, screw insertion angles, and bicortical status between the control and guide groups. The Kruskal–Wallis test was used to compare differences between the CT, bone models, and cadavers. Moreover, an additional analysis for comparison between the groups was performed using Bonferroni’s method. Statistical significance was set at *P* < 0.05.

## Results

A total of 72 screws were used in 12 cadavers, 6 in the control group, and 6 in the guide group. The six 3D-printed guides conformed well to the surface of both the printed bone model and the C1 and C2 vertebrae of the cadaver subjectively.

### Grade evaluations

The 72 screws used in the control and guide groups were evaluated for the degree of vertebral canal penetration (Table 2). A total of 24 screws were used for C1, and 48 screws for C2. The guide group (33/36, 92%) had more screws that did not breach the vertebral canal (Grade 1) than the control group (20/36, 56%) (*P* = 0.003). In the control group, 8/26 (22%) screws partially breached the vertebral canal (Grade 2a), but none in the guide group (*P* = 0.002). The overall screw penetration to the vertebral canal (Grade 2b) was 4/36 (11%) in the control group and zero in the guide group (*P* = 0.058). In the control group, 3/36 (8%) of the screws partially caused lateral breaching (Grade 3a) compared to 2/36 (6%) in the guide group (*P* = 0.575). Each group had one screw that caused the entire lateral breaching (Grade 3b) (P = 1). Overall, significantly more screws were evaluated as Grade 1 in the guide group than in the control group, and Grade 2 was not observed in the guide group.

**Table 2:** Degrees of the vertebral canal penetration (modified Zdichavsky classification).

	Control group				Guide group				P-value
	C1	C2 Cranial	C2 Caudal	Overall	C1	C2 Cranial	C2 Caudal	Overall	
**Grade 1**	5	8	7	20	12	12	9	33	0.003**
**Grade 2a**	4	4	0	8	0	0	0	0	0.002**
**Grade 2b**	3	0	1	4	0	0	0	0	0.058
**Grade 3a**	0	0	3	3	0	0	2	2	0.575
**Grade 3b**	0	0	1	1	0	0	1	1	1.000

*p < 0.05

**p < 0.01.

### Screw insertion angles

Two angles were measured for each screw and compared with the angle set as the standard (Table 3). Statistically significant differences were found in C1 right lateral and C2 caudal right lateral between the control group (*P* = 0.025) and the guide group (*P* = 0.016). No differences were found in the other positions (*P* > 0.05). The guide group represented more similar angles to the reference angles than in the control group, except for the C1 left caudal, C1 right caudal, and C2 cranial left ventral angles. The position that represented the greatest difference from the reference angle in the control group was the C2 cranial left lateral position (17.33), and the position with the smallest difference was the C1 right caudal position (0.51). The position that represented the greatest difference from the reference angle in the guide group was the C2 cranial right lateral position (12.83), and the position with the smallest difference was the C2 caudal left lateral position (0.37).

**Table 3:** Screw insertion angles.

Screw insertion position	Reference angle	Measured angle		Angle difference		
		Control group	Guide group	Reference vs Control	Reference vs Guide	P-value
**C1 left lateral**	20	17.58 (7.09–28.44)	20.54 (14.91–27.77)	2.42	0.54	.631
**C1 right lateral**	20	13.43 (7.16–22.34)	21.93 (18.62–27.17)	6.57	1.93	.025*
**C1 left caudal**	10	8.27 (1.92–12.68)	6.62 (0.67–14.42)	1.73	3.38	.337
**C1 right caudal**	10	10.51 (3.53–25.34)	8.58 (1.11–14.77)	0.51	1.42	.749
**C2 Cr left lateral**	45	27.67 (18.67–39.77)	35.43 (24.96–45.60)	17.33	9.57	.109
**C2 Cr right lateral**	45	28.36 (16.71–35.36)	32.17 (28.21–38.01)	16.64	12.83	.423
**C2 Cr left ventral**	35	33.44 (17.29–43.49)	29.11 (19.38–37.79)	1.56	5.89	.262
**C2 Cr right ventral**	35	41.15 (19.39–70.23)	33.50 (27.21–39.96)	6.15	1.50	.631
**C2 Cd left cranial**	1	5.31 (1.63–9.87)	4.47 (1.74–9.49)	4.31	3.47	.749
**C2 Cd right cranial**	1	7.20 (0.47–18.86)	4.09 (0.70–10.66)	6.20	3.09	.337
**C2 Cd left lateral**	29	29.55 (16.63–34.65)	29.37 (23.56–40.81)	0.55	0.37	.522
**C2 Cd right lateral**	29	24.67 (15.59–30.21)	32.48 (27.20–39.53)	4.33	3.48	.016*

Abbreviations: Cr, cranial; Cd, caudal.

Note: Data are presented as the mean (range) for groups. The angle difference is the absolute value of the difference between the reference and mean angles.

*p < 0.05

**p < 0.01.

### Application of bicortical screws

**Fig 6:**
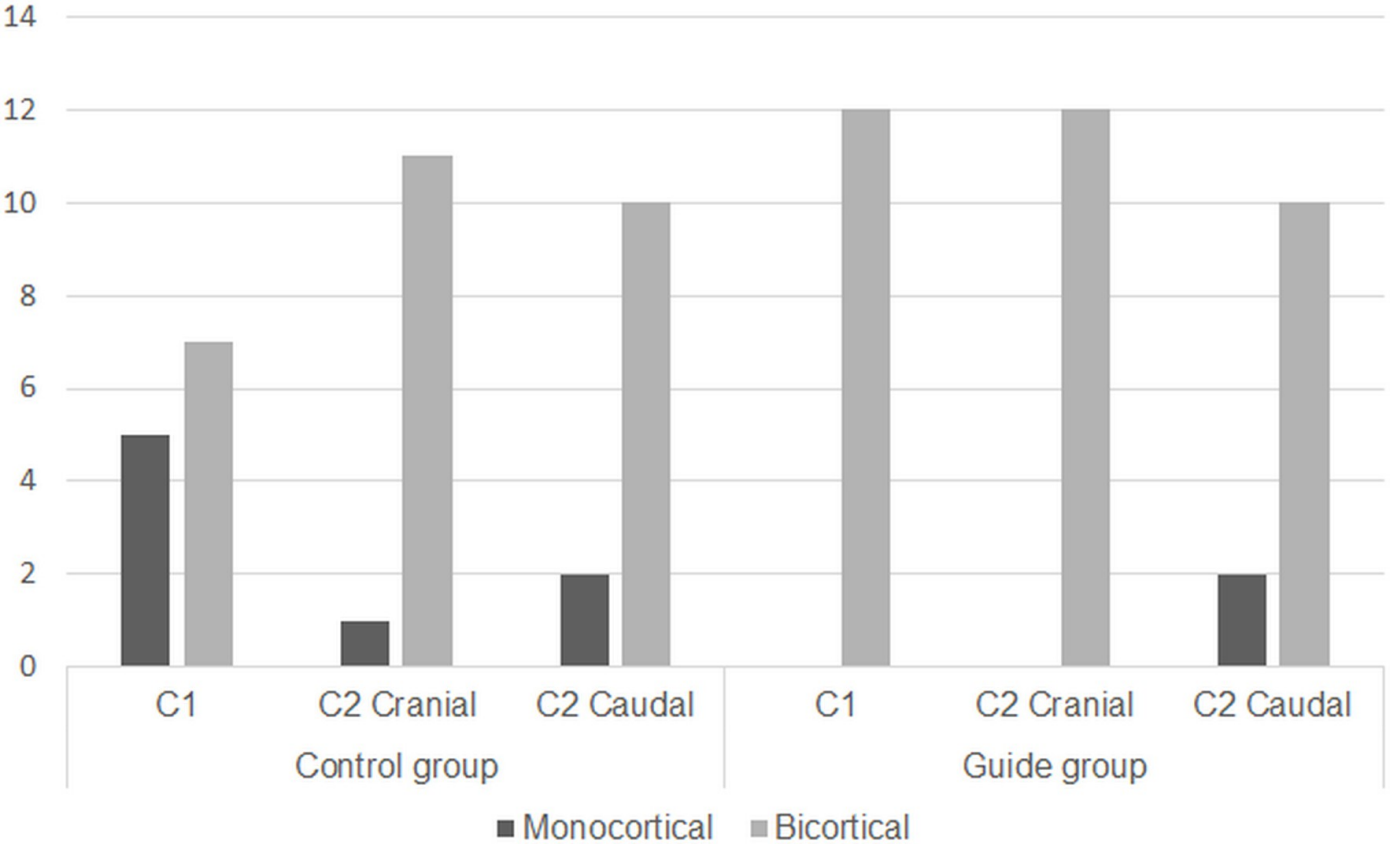
Evaluation of the bicortical screws.

Each screw was evaluated for (intended) bicortical depth via CT transverse plane (Fig 6). The number of bicortically applied screws in the control group was 28/36 (78%), and the number of monocortical screws was 5/36 (14%) in C1, 1/36 (3%) in C2 cranial, and 2/36 (6%) in C2 caudal. The number of bicortically applied screws in the guide group was 34/36 (94%), and the number of monocortical screws was 2/36 (6%) in C2 caudal. No screws were applied monocortical to C1 and C2 cranial in the guide group.

### Screw length comparisons using CT, bone models, and cadavers

The screw length was compared between the planning CT, cadavers, and bone models in the guide group (Table 4). The difference between the three values in the C1 right position was statistically significant (*P* = 0.004). Contrastingly, these three values in the other positions showed no statistical difference (*P* > 0.05). The post-analysis that determined significant differences between each group revealed more similar comparisons between the bone models and the cadavers than the comparisons with CT only at the right position of C1. No differences were found in the other positions in the paired comparisons of the three groups.

**Table 4:** Screw length in millimeters determined using CT, bone models, and cadavers in the guide group.

	C1 left	C1 right	C2 Cr left	C2 Cr right	C2 Cd left	C2 Cd right
**CT**	14.67 (14–16)	14.33 (14–16)	10.67 (10–12)	10.67 (10–12)	12.33 (12–14)	12.33 (10–14)
**Bone model**	16.33 (14–20)	17.67 (16–20)	10.33 (10–12)	11.33 (10–14)	11.33 (10–12)	12.33 (10–16)
**Cadaver**	16.33 (14–20)	17.67 (16–20)	10.33 (10–12)	10.00 (8–12)	10.33 (8–12)	11.00 (8–12)
**P-value**	0.462	0.004**	0.738	0.295	0.084	0.363
**Post hoc analysis**	N/A	Bone model, Cadaver^a^	N/A	N/A	N/A	N/A

Abbreviations: N/A, not applicable; Cr, cranial; Cd, caudal.

Note: Data presented are mean (range) for each position.

^a^Measures from the bone models and the cadavers were statistically similar compared to CT.

*p < 0.05

**p < 0.01.

## Discussion

To our best knowledge, this is the first study to compare the accuracy of a patient-specific 3D-printed drill guide versus screw application by eye using only anatomic markers and access to a bone model in AAI ventral stabilization surgery. This study performed AAI stabilization using a patient-specific guide in half the cadavers but without one in the other group. The surgeon had access to a 3D-printed bone model in both groups. The results of this study showed that the surgical accuracy was higher when the guide was used in addition to a bone model.

The comparison of the degree of vertebral canal penetration revealed that the number of screws that did not breach the vertebral canal (Grade 1) was significantly higher in the guide group 92%) than that in the control group (56%). Therefore, the guides provided greater accuracy for screw insertion and would lessen the risk of complications in clinical cases. The number of screws that were partially or fully deviated from the pedicle was 8% in the guide group and 44% in the control group. This is similar to other reports that used a patient-specific guide for AAI stabilization [15]. When patient-specific guides were applied to different vertebrae, the rate of vertebral canal penetration was 9%, 14%, and 21% [17,21,22] similar to the 8% rate in this study.

In the guide group, screws other than Grade 1, were found in 3/36 (8%) screws and all appeared at the C2 caudal position, with two of Grade 3a and one of Grade 3b. The C2 caudal position is very likely to penetrate the transverse foramen because the pedicle is anatomically very thin. Therefore, the thickness of the C2 caudal pedicle was measured with CT, and screws with a smaller diameter compared to other locations were used, where necessary (Table 1). A previous study applied a screw to penetrate the transverse foramen on one side only when the C2 caudal pedicle was too small for a 1.5 mm screw. Damaging the ipsilateral vertebral artery was possible, but no side effects were reported [15]. A study on ventral fixation of AAI stabilization reported a method of using a screw placed in the center of the caudal part of the C2 vertebral body [6]. Damaging the spinal cord is possible if the screw is directed in the vertebral canal direction. Thus, the direction of the C2 caudal screw was set to the lateral direction in this study [6].

In the control group, 7/12 (58%) screws in the C1 position penetrated the vertebral canal. Without a guide, the starting position and drill angle were determined by eye and executed by hand. The ventral cortex of C1 is relatively smooth and arched, thus slipping of the drill guide during free drilling is highly possible, resulting in a hole created in a position different from the planned position. Accurately drilling the pedicle at an intended angle by eye is difficult. Freehand drilling relies on an accurate subjective judgment of the angle despite access to the bone model. In the case of using a patient-specific guide, the guide is fixed to the surface of the bone; therefore, a drill hole can be created at the planned position, and drilling is determined by the angle set by the computer-aided design, making the pedicle drilling more reliable and accurate. This study, revealed no cases of C1 that penetrates the vertebral canal in the guide group.

No statistical significance was found, except for the two positions in the angle comparison according to the screw position. The comparison of the difference with the reference angle at a specific location revealed a more similar value in the control group with the reference value than that represented by the guide group. However, the angle to the lateral was closer to the reference value in the guide group than in the control group. This is a more significant value for the vertebral canal penetration than the angle to the other direction. Thus, the guide group did not penetrate the canal in the actual results. Moreover, the standard deviation of the guide group was less than that of the control group. Furthermore, the difference between the left and right angles was also smaller in the guide group. These results indicate that if you use a guide, you can consistently insert the screw on the left and right, and at similar angles, without significant differences and independent of the dog.

The surgical window is narrow, and the surrounding soft tissue is thick during surgery. In this study, the angles of C1 lateral and C2 ventral were applied to the safety margin, and not the reported mean projected angle [12]. When the author applied the mean projected angle, the angle of the drill guide was limited. Therefore, drilling at the correct angulations for both angles was difficult. The angle was set to minimize interference from the surrounding tissues to ensure that angulation did not deviate from the safety margin, specifically the values were set at 10° for C1 lateral and 35° for C2 ventral.

Differences from the set reference angles, even though the patient-specific guide was used, are due to the following. 1) Errors in the guide production process. During the guide production process, a difference may have occurred in the measurement reference point of the angle by making the guide through a 3D-implemented bone model. 2) A variation may have occurred by measuring the angle based on the cross-section of the CT images. 3) Some soft tissue remnants may have possibly persisted, and the difference may have occurred because the guide did not completely fit the bone surface. 4) The guide may have moved during the procedure because only one temporary pin was used to fix the C1 guide. Increasing the temporary pin of the C1 guide to two may be necessary for a more accurate surgical application.

Reportedly, the resistance of the construction to cyclic loading tends to increase by bicortically placing screws because of the greater working length and bone-implant interface [23]. During surgery, when the screw is directed to the vertebral canal, the bicortical application screw may cause spinal cord damage if the length measurement is incorrect. In some cases, the screw is applied monocortical, then the screw is fixed with a cerclage wire, and PMMA is applied to increase the fixing power [9]. In this study, the length to which the screw can be bicortically applied was measured in advance through CT, and the second length was measured using the bone model, after which the screw was applied. The rate of monocortical screw application (22%), was higher in the control group than that in the guide group (6%). All screws in the C1 and C2 cranial were bicortically applied in the guide group because a screw that penetrated the vertebral canal did not exist. Furthermore, the screw was inserted in the correct position in the guide group; unlike the control group, where the application position of the screw can be variable.

The comparison of the screw length from preoperative planning and the screw length used in the bone models and cadavers in the guide group revealed that C1 right was the only statistically significant position. A difference of 2 mm can affect the choice of screw length, and it can also affect whether it is bicortical. This study revealed that the positions with an average difference of close to 2 mm were the C1 left, C1 right, and C2 caudal left positions. This value is an average, calculated regardless of the diameter of the dog and screw, thus errors are possible. However, the C2 cranial position was approximately the same length as the other three groups. These values suggest the possibility of differences between the length determined during preoperative planning and the length of the actual screw at the C1 and C2 caudal positions. This may be related to the determined length during preoperative planning that is calculated based on the CT cross-section. The angle may also differ from the preoperative plan due to residual soft tissues and guide contraction. The comparison of the C1 and C2 caudal positions revealed fewer differences between the bone models and the cadavers than that between the CT and the cadavers. Our study suggest that a more accurate screw length can be determined if a preoperative plan based on CT is developed and a simulated operation is applied to the bone model, and then applied to the patient. Additionally, the comparison was inaccurate possibly because data analysis was based on dogs of varying sizes. Therefore, further research on dogs of similar sizes is required.

In this study, the diameter of the guide hole was made to fit the drill sleeve and not the drill bit diameter. The guide hole itself can serve as a drill sleeve if it is made with the diameter of the drill bit. However, the hole of the guide may be damaged as the drill bit rotates, or the guide may be deformed by the heat generated by the rotation. Additionally, the drill bit diameter was smaller than that of the drill sleeve; thus, the diameter of the hole decreased, thereby increasing the possibility of errors in printing the guide. The disadvantage of manufacturing the guide with the diameter of the drill sleeve is that the sleeve is away from the bone and more deep drill bits are inserted, thus the length of the drill bit may be insufficient, and the drill bit cannot penetrate the bone. As mentioned above, in this study, only one temporary pin was applied to the C1 guide, causing some guide movements when drilling. One temporary hole was made considering the size of the dogs and drill hole location, but movements were severe compared to the C2 guide that was fixed with two pins. The screw accuracy can be further improved if the guide is fixed by applying two temporary pins to C1 when manufacturing the guide in the future.

This study has several limitations as follows: 1) the study population was as small (12 dogs). However, 72 screws were evaluated and statistically significant values were derived. 2) Differences are possible in the accuracy comparison because general surgeons, not specialists, performed the surgeries. However, the guide group accuracy was very high and the use of a patient-specific guide provided evidence that even inexperienced surgeons could safely perform AAI stabilization surgery. 3) Only one investigator measured the angle; thus, the objectivity may be insufficient. 4) The operation must be performed by applying different angles for each individual. However, the operations in this study were performed at a predetermined angle to ensure consistency for experimental purposes. The angle was applied according to available safety margin data; thus, the difference in the angle of each individual did not greatly deviate. The guide was made and applied at the same angle in dogs of different sizes, but the results showed that the screws were accurately positioned within the pedicle.

## Conclusions

In conclusion, the use of a patient-specific 3D-printed drill guide for AAI ventral stabilization can improve surgical accuracy. The use of a patient-specific drill guide minimized vertebral canal penetration, allowed screw insertion at a fixed angle, and increased the fixation strength by bicortically applying the screw. Moreover, simulated surgeries using the bone models in preoperative planning enabled greater accuracy in the actual surgeries. Spinal cord damage may be minimized and stability may be enhanced compared to preoperative planning of AAI surgeries using only CT by determining the screw length using a bone model.

## Supporting information

S1 File Zdichavsky modified classification. (XLSX)

S2 File Inserted screw angle. (XLSX)

S3 File Screw length. (XLSX)
